# Hématome vulvaire massif du post-partum: à propos d'un cas à l'Hôpital Central de Yaoundé (Cameroun)

**DOI:** 10.11604/pamj.2014.19.167.5603

**Published:** 2014-10-17

**Authors:** Florent Ymele Fouelifack, Jovanny Tsuala Fouogue, Jeanne Hortence Fouedjio, Zacharie Sando, Robinson Enow Mbu

**Affiliations:** 1 Service de Gynécologie et Obstétriques de l'Hôpital Central de Yaoundé, Cameroun; 2 Département de Gynécologie et Obstétriques de la Faculté de Médecine et des Sciences Biomédicales de l'Université de Yaoundé 1, Cameroun; 3 Départerment des Sciences Morphologiques de la Faculté de Médecine et des Sciences biomédicales de l'Université de Yaoundé 1, Cameroun

**Keywords:** Hématome vulvaire, hématome puerpéral, hémorragie du post-partum, Cameroun, vulvar hematoma, postpartum hematoma, Postpartum hemorrhage, Cameroon

## Abstract

Les hématomes puerpéraux sont une cause rare d'hémorragie du post partum. Leur prise en charge adéquate nécessite une compétence et un plateau technique particulier. A notre connaissance, aucun cas n'a été publié au Cameroun. Nous rapportons le cas d'une femme de 37 ans, G_3_P_2013_, référée d'un dispensaire vers la maternité de l'hôpital Central de Yaoundé, en état de choc hémorragique survenu une heure après un accouchement facilité par des manœuvres digitales de dilatation vaginale. Elle a été prise en charge chirurgicalement pour un hématome vulvaire expansif. Ce cas nous permet d'attirer l'attention des praticiens sur la gravité et la singularité de cette pathologie hautement morbide qui pourrait être due à des manœuvres de dilatation digitale du vagin pendant le travail.

## Introduction

L'hémorragie du post-partum est la première cause de mortalité maternelle dans le monde avec une contribution variant de 18 à 50% des décès [1–5]. Les étiologies les plus fréquentes sont: l'atonie utérine, les coagulopathies, la rétention de débris placentaires, les anomalies d'insertions placentaires, la rétention des membranes fœtales et les lacérations du tractus génital [4, 5]. Parmi les causes inhabituelles, figurent les hématomes puerpéraux (encore appelés thrombus péri-génitaux) avec une fréquence de 1/1000 accouchements [6]. Ces derniers sont de diagnostic facile lorsqu'ils concernent la vulve et/ou le vagin tandis que les localisations pelviennes (retro-péritonéales) nécessitent plus de subtilité de la part des praticiens ainsi que la réalisation des examens d'imagerie [7]. Nous rapportons ici un cas d'hématome vulvaire expansif du post-partum immédiat, pris en charge chirurgicalement.

## Patient et observation

Madame BA âgée de 37 ans, G3P2013, mariée, commerçante a été référée en urgence dans notre service pour prise en charge d'une tuméfaction des lèvres droites. Le début était marqué par la survenue après l'accouchement d'un saignement vaginal de grande abondance, suivie d'une tuméfaction vulvaire (lèvres droites) très douloureuse. Devant ce tableau le personnel du dispensaire avait mis en œuvre les mesures suivantes: massage utérin, révision utérine, pose d'une voie veineuse au sérum glucosé à 5% contenant 20 unités internationales d'ocytocine à un débit non précisé. A l'arrêt du saignement vaginal une asthénie extrême ainsi qu'une pâleur cutanéo-muqueuse ont été observées, ainsi que l'apparition quelques minutes plus tard d'une tuméfaction progressive et très douloureuse des grandes lèvres droites. L'accouchement n’était pas instrumental. La patiente rapportait des manœuvres digitales de dilatation vaginale vigoureuses et douloureuses pendant toute la durée du deuxième stade du travail. Comme antécédents, la patiente a eu les ménarches à 12 ans et le premier coït à 13 ans. Elle saigne pendant 4 jours tous les à 30 jours. Sa première grossesse était gémellaire et avait abouti à un accouchement par voie basse à terme sans complication 4 années plus tôt, d'une fille et d'un garçon pesant respectivement 2,8 et 3,2 kilogrammes. Sa deuxième grossesse s'est achevée par une fausse couche spontanée sans complications à 8 semaines. Elle n'a jamais été transfusée et est du groupe sanguin A, rhésus positif. Les autres antécédents n’étaient pas contributifs. L'enquête systématique retrouvait en dehors du motif de consultation: une soif intense, une asthénie extrême et des vertiges.

**Figure 1:**
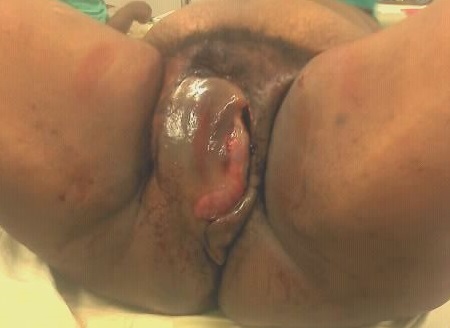
Hématome expansif disséquant la grande lèvre droite

A l'examen physique, l’état général était altéré par une pâleur et une asthénie. La température était de 37,4 degrés Celsius, le pouls de 96 pulsations par minute, la tension artérielle de 82/66 millimètres de mercure et la fréquence respiratoire de 24 cycles par minutes. Les conjonctives étaient pâles. L'abdomen était souple et indolore. Les bruits hydro-aériques étaient présents et l'utérus, bien contracté. L'inspection des organes génitaux externes révélait une tuméfaction bleutée de l'hémi vulve droite atteignant le pli inguinal en dehors, le grand pli fessier en arrière, le mont de venus en avant. En dedans, la masse refoulait la petite lèvre et faisait saillie dans la paroi vaginale d'où s’écoulait un fin filet de sang à travers une déchirure intéressant la musculeuse et la muqueuse (Figure 1). La peau était luisante, sous forte tension et présentait des plages de dermabrasions. La palpation était extrêmement douloureuse et rendait difficile l'exploration de la masse sans anesthésie. Son grand diamètre était antéropostérieur et mesurait 17 centimètres tandis que son petit diamètre, transversal mesurait 12 centimètres. Ces dimensions augmentaient assez rapidement tout comme la douleur rapportée par la patiente.

**Figure 2:**
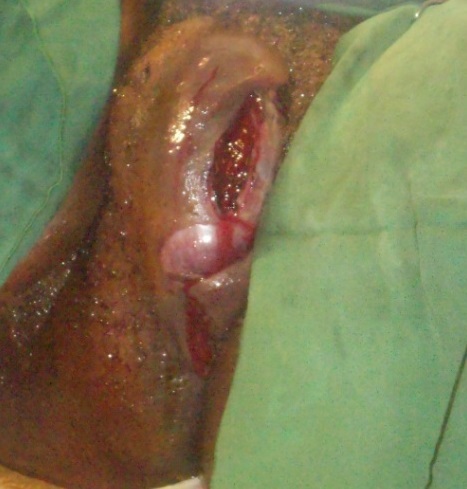
Hématome situé entre les plans du périnée

**Figure 3:**
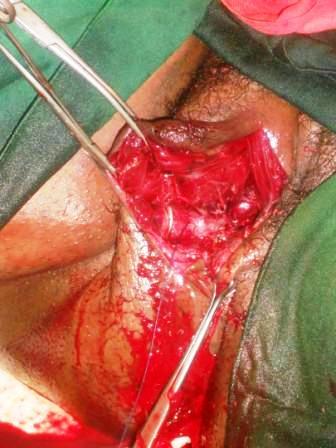
Cavité résiduelle après évacuation de l'hématome

**Figure 4:**
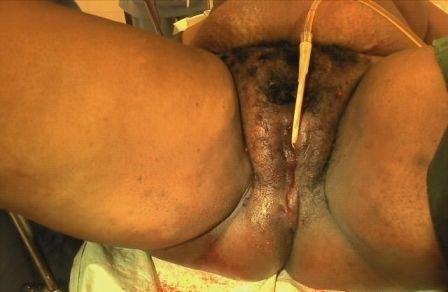
Aspect de la vulve à la fin chirurgie

L'examen des membres montrait une pâleur des extrémités sans autres anomalies. Nous avons posé le diagnostic d'hématome vulvaire expansif du post partum immédiat compliqué d'anémie sévère. Une exploration chirurgicale sous anesthésie générale en vue de réaliser l'hémostase a aussitôt été indiquée ainsi qu'une transfusion de 2000 millilitres de sang total. En dehors du taux d'hémoglobine de 4,9 grammes/décilitre, le bilan préopératoire était sans particularité. Les trouvailles per-opératoires (Figure 2) étaient: un hématome de 800 millilitres situé entre les plans superficiel et profond du périnée et un saignement actif provenant des artères périnéales superficielle et profonde (Figure 3). Après avoir évacué l'hématome, nous avons réalisé avec succès plusieurs points de suture hémostatique en « X » à l'aide d'un fil de marque Vicryl^®^ 0. Le capitonnage de la cavité résiduelle ayant ensuite été fait aisément. Enfin, la suture de la déchirure vaginale homolatérale a été réalisée ainsi qu'une compression pendant 12 heures par un tampon vaginal (Figure 4). Aucun saignement n'a été noté après l'ablation dudit tampon. Dans les suites opératoires le protocole de douches vaginales antiseptiques n'a pas été correctement réalisé. L’évolution était marquée par la suppuration de la plaie motivant un renforcement des soins post – opératoires et un prolongement du séjour et la patiente, qui a quitté l'hôpital au neuvième jour post – opératoire. Trois semaines plus tard l'anatomie vulvo-vaginale était macroscopiquement normale.

## Discussion

L'hématome vulvaire (HV) est une entité clinique qui se rencontre soit dans le contexte obstétrical. En dehors de la grossesse et du post partum les circonstances les diverses voire insolites ont été rapportés: traumatisme par chute à califourchon et coït [8–10]. La fréquence de l'Hématome Vulvaire Puerpéral (HVP) varie selon que son volume est faible (1 cas pour 700 accouchements) ou important (1 cas pour 4 000 accouchements) (Sadoul cité par Riethmuller) [6]. Les facteurs de risques de l'HVP reconnus dans la littérature sont: la primiparité, l'extraction fœtale instrumentale, la pré-éclampsie, les grossesses gémellaires, la macrosomie et les varices vulvo-vaginales [6]. Chez notre patiente est la réalisation digitale de la dilatation vaginale pendant tout le deuxième stade du travail se rapprocherait par son mécanisme, d'un accouchement par forceps. Bien que n'ayant pas encore fait l'objet ni d’études scientifiques ni de recommandations pour la clinique, cette pratique empiriques est cependant très répandue et quasiment systématique dans notre contexte. Il serait judicieux de se pencher de façon scientifique sur la pratique et de procéder le cas échéant à une sensibilisation du personnel des salles d'accouchement sur ses effets potentiellement néfastes.

Comme le rapporte la littérature, le tableau clinique chez notre patiente était dominé par la survenue d'une douleur et d'une tuméfaction généralement unilatérale quelques instants ou aussitôt après l'accouchement [6, 11]. Une déchirure vaginale homolatérale et une atonie utérine étaient associées et expliquaient probablement l'abondance du saignement observé ainsi que le choc hémorragique qui s'en est suivi. La littérature rapporte plusieurs formes topographiques d'hématomes puerpéraux: l'hématome vulvaire, l'hématome vaginal, l'hématome vulvo-vaginal, et l'hématome pelvi -génital ou sous péritonéal [6].

Le traitement de fond repose sur l'hémostase et la correction des perturbations hémodynamiques et les mesures adjuvantes incluent l'antibioprophylaxie, et l'antalgie [6–9, 11]. L'hémostase peut se faire toute seule par compression de la lésion vasculaire par le caillot constituant l'hématome [6, 11]. Dans les cas contraires un geste hémostatique s'impose et consiste dans un premier temps en une exploration chirurgicale du site de l'hématome (incision – évacuation de l'hématome - sutures hémostatiques en « X » drainage – méchage – tamponnement vaginal sur une sonde urinaire à demeure). Tel était le cas chez notre patiente. La principale difficulté est de localiser le vaisseau hémorragique dans un champ opératoire masqué par l’écoulement de sang [6]. Dans les suites opératoires, l’étape la plus délicate est l'ablation des mèches qui peut donner lieu à une récidive du saignement. En cas d’échec immédiat de l'hémostase chirurgicale ou de récidive de l'hémorragie l’étape suivante est la ligature des artères utérines [6, 11]. Si celle-ci échoue, l'embolisation sélective des artères pudentale et glutéale inférieure représente est l'ultime recours thérapeutique; sa mise en œuvre reste cependant limitée par la nécessité d'un plateau technique et d'une équipe de radiologie interventionnelle expérimentée [6, 10, 11]. Dans notre contexte cette technologie n'est pas disponible et à l'ablation du tampon nous n'avons pas noté de récidive de l'hémorragie. Trois semaines après l'intervention l'anatomie vulvo – vaginale était macroscopiquement normale et la patiente a repris l'activité sexuelle six semaines après la chirurgie.

## Conclusion

La survenue de cet hématome puerpéral devrait attirer l'attention des accoucheurs sur la pratique intempestive de la dilatation digitale de l'orifice vulvo-vaginal pendant le travail.
